# Comparison of Hydroxyapatite/Poly(lactide-co-glycolide) and Hydroxyapatite/Polyethyleneimine Composite Scaffolds in Bone Regeneration of Swine Mandibular Critical Size Defects: In Vivo Study

**DOI:** 10.3390/molecules27051694

**Published:** 2022-03-04

**Authors:** Momir Stevanovic, Dragica Selakovic, Miroslav Vasovic, Biljana Ljujic, Suzana Zivanovic, Milos Papic, Marko Zivanovic, Nevena Milivojevic, Milica Mijovic, Sasa Z. Tabakovic, Vukoman Jokanovic, Aleksandra Arnaut, Pavle Milanovic, Nemanja Jovicic, Gvozden Rosic

**Affiliations:** 1 Department of Dentistry, Faculty of Medical Sciences, University of Kragujevac, 34000 Kragujevac, Serbia; momirstevanovic7@gmail.com (M.S.); miki_vasovic@yahoo.com (M.V.); suzanazivanovic91@yahoo.com (S.Z.); milos_papic@live.com (M.P.); sandra11_92@yahoo.com (A.A.); pavle11@yahoo.com (P.M.); 2 Department of Maxillofacial Surgery, Medical Faculty Pristina in Kosovska Mitrovica, 38220 Kosovska Mitrovica, Serbia; sasataba@yahoo.com; 3 Department of Physiology, Faculty of Medical Sciences, University of Kragujevac, 34000 Kragujevac, Serbia; dragica984@gmail.com (D.S.); grosic@medf.kg.ac.rs (G.R.); 4 Department of Genetics, Faculty of Medical Sciences, University of Kragujevac, 34000 Kragujevac, Serbia; bljujic74@gmail.com; 5 Department of Science, Institute for Information Technologies Kragujevac, University of Kragujevac, 34000 Kragujevac, Serbia; zivanovicmkg@gmail.com (M.Z.); nevena_milivojevic@live.com (N.M.); 6 Institute of Pathology, Faculty of Medicine, University in Priština, 38220 Kosovska Mitrovica, Serbia; milica.mijovic@med.pr.ac.rs; 7 Department of Atomic Physics, Vinca Institute of Nuclear Sciences, 11000 Belgrade, Serbia; vukoman@vin.bg.ac.rs; 8 Department of Histology and Embryology, Faculty of Medical Sciences, University of Kragujevac, 34000 Kragujevac, Serbia

**Keywords:** composite scaffolds, polyethyleneimine, hydroxyapatite ceramics, poly(lactide-co-glycolide), mandibular defect

## Abstract

Reconstruction of jaw bone defects present a significant problem because of specific aesthetic and functional requirements. Although widely used, the transplantation of standard autograft and allograft materials is still associated with significant constraints. Composite scaffolds, combining advantages of biodegradable polymers with bioceramics, have potential to overcome limitations of standard grafts. Polyethyleneimine could be an interesting novel biocompatible polymer for scaffold construction due to its biocompatibility and chemical structure. To date, there have been no in vivo studies assessing biological properties of hydroxyapatite bioceramics scaffold modified with polyethyleneimine. The aim of this study was to evaluate in vivo effects of composite scaffolds of hydroxyapatite ceramics and poly(lactide-co-glycolide) and novel polyethyleneimine on bone repair in swine’s mandibular defects, and to compare them to conventional bone allograft (BioOss). Scaffolds were prepared using the method of polymer foam template in three steps. Pigs, 3 months old, were used and defects were made in the canine, premolar, and molar area of their mandibles. Four months following the surgical procedure, the bone was analyzed using radiological, histological, and gene expression techniques. Hydroxyapatite ceramics/polyethyleneimine composite scaffold demonstrated improved biological behavior compared to conventional allograft in treatment of swine’s mandibular defects, in terms of bone density and bone tissue histological characteristics.

## 1. Introduction

Trauma, craniofacial deformities, tumors, or infections in the maxillofacial area may result in significant facial deformities and dysfunctions with significant decrease in the quality of life of patients [1]. Reconstruction of the facial and jaw bone defects is a significant problem in oral and maxillofacial surgery due to specific esthetic and functional requirements [1]. Extensive clinical research on bone reconstruction with standard autograft and allograft transplantation procedures, although with good clinical results, demonstrated significant limitations related to the problems of donor availability, donor site morbidity, supply difficulties, pathogen transfer, and immune system rejection [2,3]. To overcome drawbacks of conventional bone regeneration procedures, extensive research on bone tissue engineering (BTE) with bio-mimicking inspired bone replacement materials, has been performed in the last three decades. BTE comprises interaction of several key factors in bone regeneration: a biocompatible scaffold serving as an extracellular matrix in which osteogenic cells form bone tissue matrix, morphogenic signals, and proteins that help to direct the cells differentiation to osteogenic phenotype and sufficient vascular supply [4]. Bone substituents or scaffolds are biodegradable materials used to fill the bone defect and serve as artificial extracellular matrixes in order to induce bone formation until restoration of its biomechanical properties [5]. In order to promote natural bone formation, scaffolds should possess adequate mechanical strength during tissue healing as well as certain surface topography characteristics and structural porosity in order to achieve good osteoconductivity, osteoinductivity, and osteointegrativity [6,7,8]. Adequate chemical, mechanical, and structural properties are the key points in promoting cellular adhesion, cell spreading, differentiation, and proliferation [7,8]. A requirement for biomaterials that can demonstrate complex functions leads to the development of a new generation of biomaterials characterized by nanofibers [9,10].

Bone tissue is a natural composite built as a mixture of organic (collagen fibers) and inorganic substance (hydroxyapatite crystals). Thus, composite scaffolds combining advantages of biodegradable polymers with bioceramics to induce bone repair, have been extensively studied. Biocomposites hydroxyapatite ceramics (HAP) are highly biocompatible and resemble the natural bone structure and its mechanical and osteoconductive properties are enhanced with thin biodegradable polymer coating [8]. Recent studies found that composite scaffolds composed of HAP and poly(lactide-co-glycolide) (PLGA) had numerous desirable characteristics such as excellent biocompatibility, adequate mechanical properties, and desirable structural requirements (>50% total porosity and >100 μm average pore size) for successful bone repair [8,11]. In vivo studies with HAP/PLGA scaffolds showed complete repair of critical size defect in rabbit’s calvaria, large defect of rabbit’s ulna, as well as critical size mandibular defect in mini pigs achieved in 12 weeks [2,6,9,10]. With regard to biocompatible polymers, polyethyleneimine (PEI) could be an interesting novel biopolymer for scaffold construction due to its biocompatibility, chemical structure, and cellular activity [12,13,14]. PEI is a poly-cationic polymer with high density of protonated secondary amines commonly involved in gene-activated scaffolds as a gene delivery agent [12]. Having in mind excellent biological properties of PEI, it could be used as a promising novel coating substance in composite scaffolds with HAP. “The main purpose of this study was to introduce novel bone scaffold based on non-stoichiometric HAP with surface modification based on PEI deposition (HAP/PEI), and to evaluate the effect on bone repair in swine’s mandibular defects. Additionally, the obtained results for radiological and histological analysis were compared with those of the well-known commercial products composite scaffold HAP/PLGA (ALBO OS) and xenograft Bio-Oss^®^ Geistlich (Wolhusen, Switzerland)”.

## 2. Results

### 2.1. Bone Density

**Figure 1:**
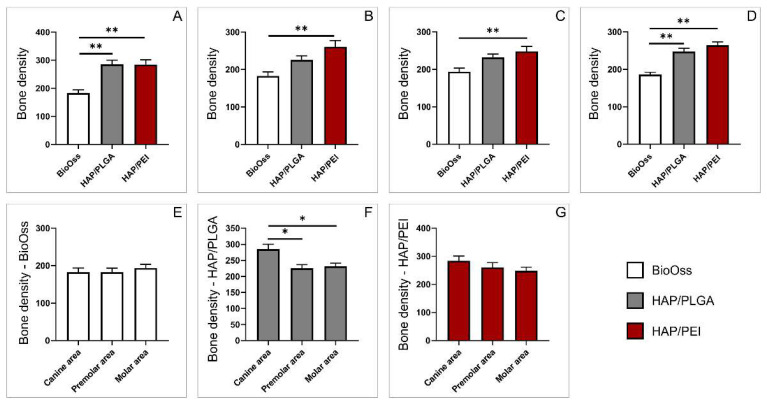
Radiological assessment of bone density obtained by CBCT: (**A**) canine area, (**B**) premolar area, (**C**) molar area, (**D**) whole mandibula, (**E**) BioOss graft, (**F**) HAP/PLGA graft, (**G**) HAP/PEI graft. The values are mean ± standard error of the mean (SEM), * denotes a significant difference *p* < 0.05, ** denotes a significant difference *p* < 0.01.

Radiological analysis, done by CBCT, demonstrated that in the canine area bone density was significantly lower in the BioOss group compared to HAP/PLGA and HAP/PEI groups (Figure 1A, F = 15.557, *p* = 0.0006). In two other investigated regions, premolar and molar, bone density was significantly lower in BioOss-treated animals compared to HAP/PEI group (Figure 1B,C, F = 8.726, *p* = 0.002, F = 6.553, *p* = 0.008 respectively) while there were no differences compared to HAP/PLGA group. Regardless of localization, our results showed that bone density was significantly lower in the BioOss group compared to HAP/PLGA and HAP/PEI groups (Figure 1D, F = 15.545, *p* < 0.0001).

Individual analyses of each graft material demonstrated that there were no differences in bone density in BioOss group between three investigated mandibular regions (Figure 1E, F = 0.359). The same result was obtained in HAP/PEI group (Figure 1G, F = 1.294), while in the HAP/PLGA group, bone density was significantly higher in canine area compared to premolar and molar areas (Figure 1F, F = 7.185, *p* = 0.001, *p* = 0.002 respectively).

### 2.2. Number of Cells in the Investigated Regions

**Figure 2:**
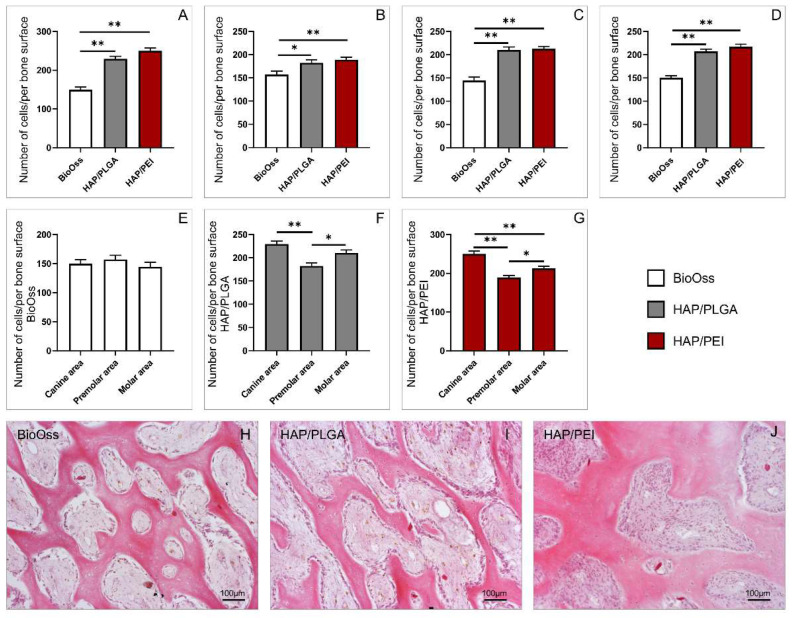
Number of cells/per bone surface: (**A**) canine area, (**B**) premolar area, (**C**) molar area, (**D**) whole mandibula, (**E**) BioOss graft, (**F**) HAP/PLGA graft, (**G**) HAP/PEI graft; representative images of H&E staining on paraffin-embedded sections (original magnification × 20): (**H**) BioOss graft, (**I**) HAP/PLGA graft, (**J**) HAP/PEI graft. The values are mean ± standard error of the mean (SEM), * denotes a significant difference *p* < 0.05, ** denotes a significant difference *p* < 0.01.

Pathohistological analysis of hematoxylin and eosin staining was used to identify and evaluate cellularity of the investigated regions of the mandibula. Obtained results demonstrated that the number of cells was significantly lower in the BioOss group compared to HAP/PLGA and HAP/PEI groups in canine, premolar, and molar areas (Figure 2A–C; F = 56.150, *p* < 0.0001, F = 6.890, *p* = 0.02, *p* = 0.003, F = 35.835, *p* < 0.0001 respectively). Moreover, in whole mandibula, regardless of the region, the number of cells was significantly lower in BioOss-treated animals (Figure 2D, F = 59.383, *p* < 0.0001). There were no observed differences in the number of cells between HAP/PLGA and HAP/PEI groups. Analysis of mandibular bone tissue treated with BioOss demonstrated that there were no differences in cellularity with regard to the investigated region (Figure 2E, F = 0.762). On the other hand, in HAP/PLGA group, the number of cells was significantly lower in the premolar area compared to canine and molar areas (Figure 2F, F = 13.212, *p* < 0.0001, *p* = 0.012 respectively). Similarly, in HAP/PEI group, the number of cells was significantly lower in the premolar area compared to canine and molar, while it was significantly higher in the canine area compared to the molar area (Figure 2G, F = 24.791, *p* < 0.0001, *p* = 0.0003, *p* = 0.03 respectively).

### 2.3. Immunoreactivity to Osteocalcin

**Figure 3:**
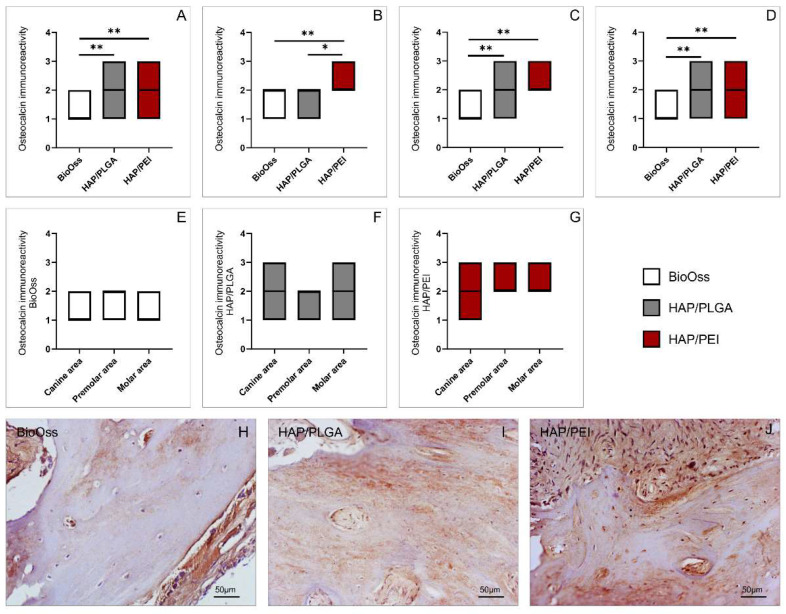
Immunoreactivity to osteocalcin: (**A**) canine area, (**B**) premolar area, (**C**) molar area, (**D**) whole mandibula, (**E**) BioOss graft, (**F**) HAP/PLGA graft, (**G**) HAP/PEI graft; representative images of H&E staining on paraffin-embedded sections (original magnification × 20): (**H**) BioOss graft, (**I**) HAP/PLGA graft, (**J**) HAP/PEI graft. Boxes represents interquartile ranges. Thick horizontal line within a box represents a median. The values are mean ± standard error of the mean (SEM), * denotes a significant difference *p* < 0.05, ** denotes a significant difference *p* < 0.01.

Immunohistochemical analysis of mandibular bone tissue demonstrated that the immunoreactivity to osteocalcin was significantly lower in the BioOss group compared to HAP/PEI in canine, premolar, and molar regions (Figure 3A, *p* = 0.0005, Figure 3B, *p* = 0.0001, Figure 3C, *p* < 0.0001 respectively). In canine and molar areas, immunoreactivity to osteocalcin was also significantly lower in the BioOss group compared to the HAP/PLGA group (Figure 3A, *p* = 0.0063 and Figure 3C *p* = 0.0004), while in the premolar area, immunoreactivity in HAP/PLGA was significantly lower compared to the HAP/PEI group (Figure 3B, *p* = 0.042). Analysis of whole mandibula, regardless of a particular region, showed that immunoreactivity was significantly lower in the BioOss group compared to HAP/PLGA, as well as HAP/PEI groups (Figure 3D, *p* < 0.0001). Separate analysis of used graft materials demonstrated that there were no significant differences in immunoreactivity to osteocalcin between canine, premolar, and molar regions (Figure 3E–G; respectively).

### 2.4. Collagen Deposition in the Investigated Regions

**Figure 4:**
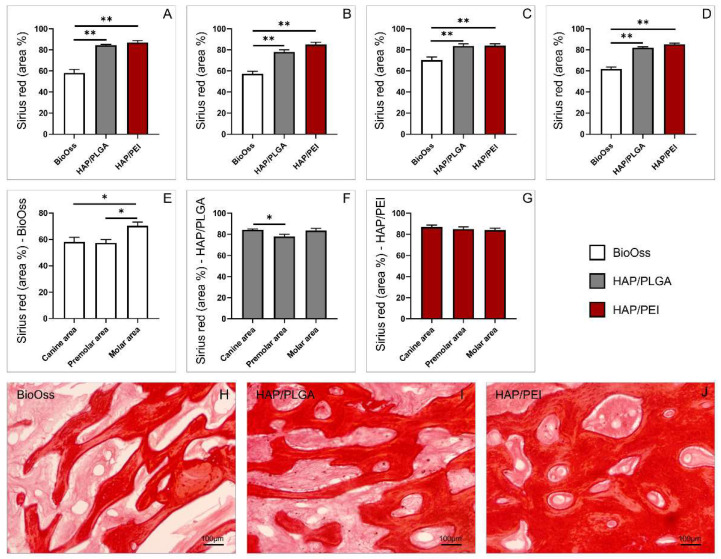
Collagen deposition in the newly formed bone tissue: (**A**) canine area, (**B**) premolar area, (**C**) molar area, (**D**) whole mandibula, (**E**) BioOss graft, (**F**) HAP/PLGA graft, (**G**) HAP/PEI graft; Representative images of H&E staining on paraffin-embedded sections (original magnification × 20): (**H**) BioOss graft, (**I**) HAP/PLGA graft, (**J**) HAP/PEI graft. The values are mean ± standard error of the mean (SEM), * denotes a significant difference *p* < 0.05, ** denotes a significant difference *p* < 0.01.

In order to evaluate parameters of the new bone formation, we used selective histochemical technique Picrosirius red. Pathohistological analysis demonstrated that the amount of collagen in the newly created bone was significantly lower in BioOss grafts compared to HAP/PLGA and HAP/PEI in canine, premolar, and molar regions (Figure 4A–C; F = 47.642, *p* < 0.0001, F = 40.952, *p* < 0.0001, F = 11.757, *p* = 0.0005, *p* = 0.0003 respectively). The same trend was also observed in whole mandibula, regardless of the graft position (Figure 4D, F = 80.445 *p* < 0.0001). Individual analysis of used graft materials demonstrated that in the BioOss group, the collagen deposition was significantly higher in the molar area compared to canine and premolar (Figure 4E, F = 6.082 *p* = 0.016, *p* = 0.01 respectively). In HAP/PLGA group, the amount of collagen was significantly higher when the graft was placed in the canine region compared to premolar (Figure 4F, F = 4.110, *p* = 0.037), while in the HAP/PEI group there were no differences between the three investigated graft positions (Figure 4G, F = 0.600).

### 2.5. Expression of Genes Involved in Bone Remodeling

In order to investigate the expression of genes which are responsible for bone remodeling and metabolism, osteocalcin, osteoprotegerin (OPG), and receptor activator of NF-κB ligand (RANKL) genes, we used RT PCR analysis.

**Figure 5:**
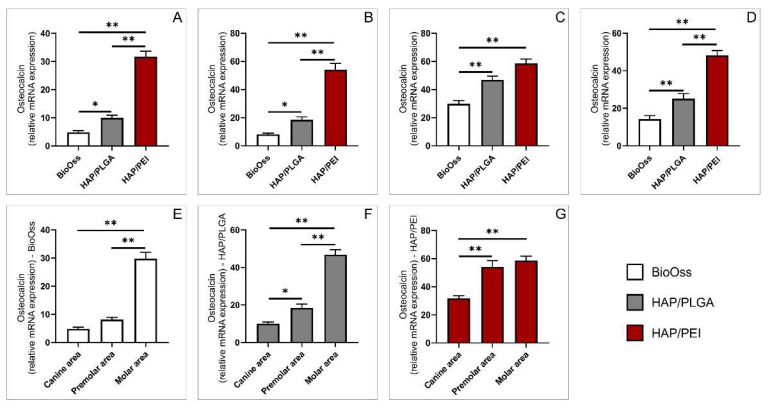
Relative expression of osteocalcin gene: (**A**) canine area, (**B**) premolar area, (**C**) molar area, (**D**) whole mandibula, (**E**) BioOss graft, (**F**) HAP/PLGA graft, (**G**) HAP/PEI graft. The values are mean ± standard error of the mean (SEM), * denotes a significant difference *p* < 0.05, ** denotes a significant difference *p* < 0.01.

The relative osteocalcin gene expression was significantly lower in the BioOss group compared to HAP/PLGA and HAP/PEI groups in all three investigated mandibular regions, canine, premolar, and molar. Similarly, HAP/PLGA-treated animals had significantly lower relative osteocalcin gene expression compared to HAP/PEI in canine, and premolar areas (Figure 5A, *p* = 0.016, *p* < 0.0001, *p* = 0.003, Figure 5B, *p* = 0.010, *p* < 0.0001, *p* = 0.006, Figure 5C, *p* = 0.007, *p* < 0.0001 respectively). The similar trends were observed in whole mandibula, regardless of the graft position (Figure 5D, *p* = 0.005, *p* < 0.0001, *p* < 0.0001 respectively). Separate analysis of graft materials showed that relative osteocalcin gene expression was significantly higher in BioOss in the molar region compared to canine and premolar regions (Figure 5E, *p* < 0.0001, *p* = 0.0003). In HAP/PLGA group, relative osteocalcin gene expression was significantly lower in the canine region compared to premolar and molar as well, while in the premolar area it was significantly lower compared to molar (Figure 5F, *p* = 0.015, *p* < 0.0001, *p* = 0.004 respectively). Analysis of HAP/PEI material showed significantly lower gene expression in canine region compared to premolar and molar regions (Figure 5G, *p* = 0.0008, *p* < 0.0001 respectively).

**Figure 6:**
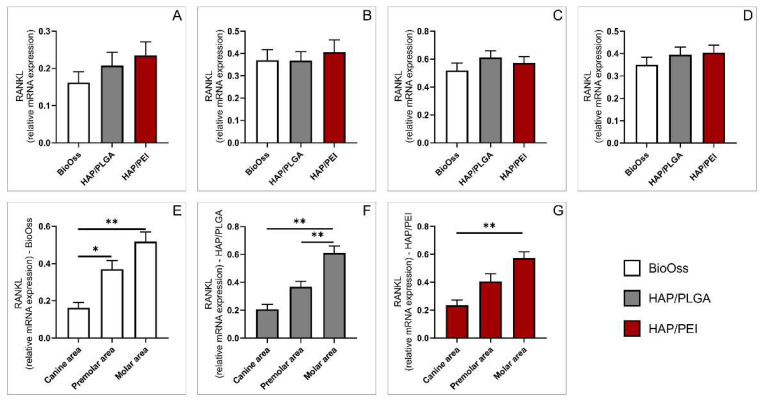
Relative expression of RANKL gene: (**A**) canine area, (**B**) premolar area, (**C**) molar area, (**D**) whole mandibula, (**E**) BioOss graft, (**F**) HAP/PLGA graft, (**G**) HAP/PEI graft. The values are mean ± standard error of the mean (SEM), * denotes a significant difference *p* < 0.05, ** denotes a significant difference *p* < 0.01.

We also analyzed relative expression of gene coding receptor activator of nuclear factor kappa-Β ligand (RANKL). No significant differences were observed in canine, premolar, and molar regions (Figure 6A–C). Similarly, there were no differences between the investigated materials in the whole mandibular bone (Figure 6D). Particular analysis demonstrated that in the BioOss group, relative RANKL gene expression was significantly lower in the canine region compared to premolar and molar areas (Figure 6E, *p* = 0.017, *p* < 0.0001 respectively). In HAP/PLGA-treated animals, expression was significantly lower in the canine area compared to molar, and also, in the premolar area compared to molar (Figure 6F, *p* < 0.0001, *p* = 0.022 respectively). Similarly, in the HAP/PEI group, relative RANKL expression was significantly lower in canine area compared to molar (Figure 6G, *p* < 0.0001).

**Figure 7:**
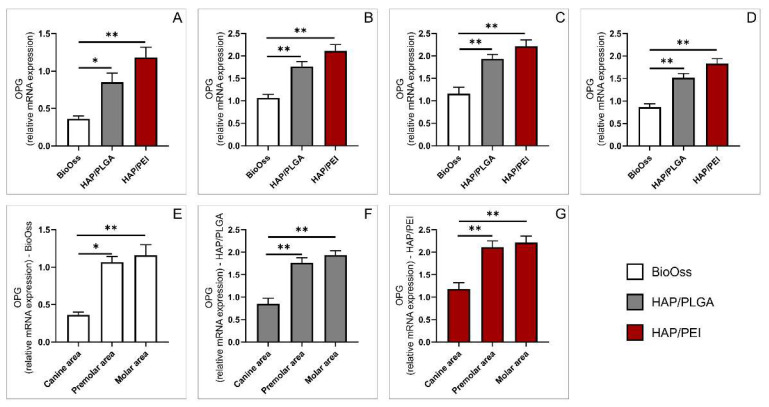
Relative expression of OPG gene: (**A**) canine area, (**B**) premolar area, (**C**) molar area, (**D**) whole mandibula, (**E**) BioOss graft, (**F**) HAP/PLGA graft, (**G**) HAP/PEI graft. The values are mean ± standard error of the mean (SEM), * denotes a significant difference *p* < 0.05, ** denotes a significant difference *p* < 0.01.

We also analyzed relative expression of gene-coding regulatory factor OPG. In all three investigated regions, we demonstrated that relative OPG gene expression was significantly lower in the BioOss groups compared to HAP/PLGA and HAP/PEI (Figure 7A, *p* = 0.02, *p* < 0.0001, Figure 7B, *p* = 0.001, *p* < 0.0001, Figure 7C, *p* = 0.0025, *p* < 0.0001 respectively). This trend was also observed in the whole mandibular bone (Figure 7D, *p* < 0.0001). Particular material analysis demonstrated that the BioOss graft localized in the canine region was related to significantly lower relative OPG gene expression compared to premolar and molar regions (Figure 7E, *p* = 0.010, *p* = 0.0008 respectively). Similarly, this trend was also observed in other two tested materials, HAP/PLGA and HAP/PEI (Figure 7F, *p* = 0.0005, *p* < 0.0001, Figure 7G *p* = 0.0012, *p* = 0.0001 respectively).

**Figure 8:**
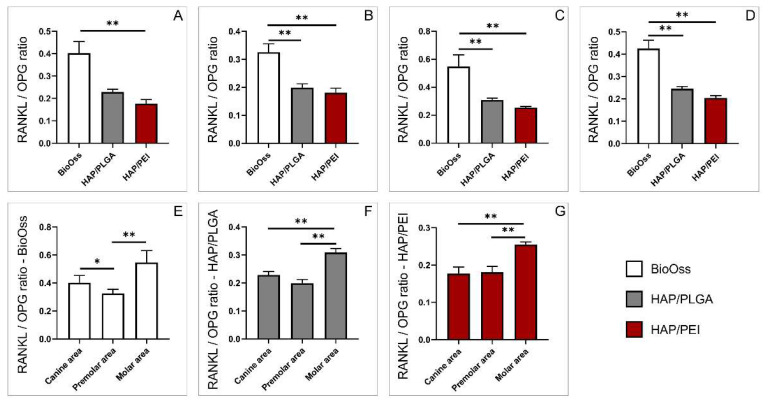
The RANKL/OPG ratio: (**A**) canine area, (**B**) premolar area, (**C**) molar area, (**D**) whole mandibula, (**E**) BioOss graft, (**F**) HAP/PLGA graft, (**G**) HAP/PEI graft. The values are mean ± standard error of the mean (SEM), * denotes a significant difference *p* < 0.05, ** denotes a significant difference *p* < 0.01.

Following gene expression analysis, we further calculated RANKL/OPG ratio, the determinant of physiological balance of bone formation and turnover [15,16,17,18]. We demonstrated that RANKL/OPG ratio was significantly higher in the BioOss group compared to HAP/PEI in all investigated regions, as well as compared to HAP/PLGA in premolar and molar regions (Figure 8A, *p* = 0.0027, Figure 8B, *p* = 0.0115, *p* = 0.0001, Figure 8C, *p* = 0.0012, *p* < 0.0001 respectively). The same trend was observed in the mandibular bone regardless of graft position (Figure 8D, *p* < 0.0001, *p* < 0.0001 respectively). Analysis of individual bone graft materials demonstrated that in the BioOss group, RANKL/OPG ratio was significantly higher in the canine compared to the premolar and lower in the premolar area compared to the molar region (Figure 8E, *p* = 0.032, *p* = 0.0016 respectively). In HAP/PLGA as well as HAP/PEI groups, the RANKL/OPG ratio was significantly higher in the molar area compared to canine and premolar regions (Figure 8F, *p* = 0.0046, *p* < 0.0001, Figure 8G, *p* = 0.0008, *p* = 0.0003 respectively).

## 3. Discussion

Reconstruction of the facial and jaw bone defects present a significant clinical problem in oral and maxillofacial surgery, mainly due to the specific esthetic and functional requirements [1]. Bone replacement grafts present a structural framework for clot development, maturation, and remodeling that ultimately leads to bone formation in osseous defects [19]. The aim of our study was to evaluate in vivo, biological properties and effects of HAP/PLGA and HAP/PEI composite scaffolds on bone repair in swine’s mandibular defects and to compare them to conventional widely used bone allograft BioOss. For that purpose, first we investigated density of the mandibular bone using CBCT technique. We used this parameter as an indicator of healing of the bone defect and embedding the graft to graft-bone complex. Our results presented in Figure 1 demonstrated that composite scaffolds, HAP/PEI in particular, induced significantly higher bone density compared to BioOss in all investigated mandibular regions. These results indicated more comprehensive bone development and bone-graft integration which occurred in the HAP/PEI group of animals, were further dissected using histological methods.

Bone formation in grafting procedures involves one or more of the following essential biological mechanisms of bone regeneration: osteogenesis, osteoinduction, osteoconduction, and osteointegration [20]. Osteogenesis or bone formation presents transformation of a pre-existing mesenchymal tissue into bone tissue [21]. In the context of bone grafting, osteogenesis presents the “osteogenic” potential of the graft or the ability of donor graft osteoprogenitor cells to proliferate and differentiate to osteoblasts and to develop the new bone [20,22,23]. Histological analysis demonstrated that natural remodeling events and new bone formation were present in all three investigated materials. There were also no signs of inflammation. Inflammatory cells were not present in the examined material and animals did not express any clinical signs of inflammation following the treatment, as well in the later stages of the experiment. These findings demonstrate that investigated materials have respectable biocompatibility, which was previously evaluated for similar compounds under different therapeutic implications [24,25,26,27]. As presented in Figure 2, the number of cells was significantly higher in HAP/PLGA and HAP/PEI composite scaffolds compared to BioOss. In BioOss group we observed fragments of newly-formed bone covered by osteoblasts with underlying osteoid. However, in HAP/PLGA and particularly in HAP/PEI specimens, newly formed bone tissue was remodeled into mature cancellous or compact bone appearing as lamellar bone structure. Cellular make-up in HAP/PEI-treated animals consists mainly of active osteoblasts and osteocytes residing in lacunae of bone tissue. Furthermore, residues of bone substitute material were very rare in this group compared to BioOss-treated animals. This finding indicates that HAP/PEI has the potential of progressive transformation into vital new bone which requires shorter healing period, and taking into account its already demonstrated favorable properties, its usage as a bone graft material seems promising [28]. Further, histological analysis of collagen deposition, presented in Figure 4, demonstrated significantly higher values in HAP/PLGA and HAP/PEI composite scaffolds compared to BioOss, although there was no difference in the amount of collagen between HAP/PLGA and HAP/PEI groups. We further investigated, by immunohistochemical and genetical approach, osteocalcin, the most abundant noncollagenous protein in bone tissue, produced by osteoblasts. As presented in Figure 3, immunoreactivity to osteocalcin was significantly higher in the HAP/PEI group compared to HAP/PLGA and BioOss. These results were further supported by gene expression analysis (presented in Figure 5) which clearly demonstrated significantly higher expression of osteocalcin gene in mandibular bones treated with HAP/PEI. Interestingly, it seems that osteocalcin gene expression is also dependent on the localization of the graft since it was significantly higher in the molar region compared to premolar and canine, in all three materials. Osteocalcin is a molecule partially responsible for the regulation of bone mineral deposition, and its expression can serve as a marker of mineralized matrix formation [29,30,31]. Therefore, the ability of composite scaffolds to induce osteoblasts to produce more osteocalcin may be an indicator of an enhanced rate of mineralized bone matrix formation [32]. In a recent study, Moriishi et al. demonstrated that osteocalcin is required for bone quality and strength by adjusting the alignment of biological apatite crystallites (BAp) parallel to collagen fibrils [33,34,35]. This role of osteocalcin further supports our findings and corresponds to the observed differences in bone architecture in the HAP/PEI group in particular. We also investigated gene expression of RANKL and OPG, molecules with pivotal role in bone remodeling and resorption. The RANKL/OPG system regulates functions of bone cells by controlling the osteoclastogenesis and bone remodeling. RANKL, produced by osteoblasts and osteocytes stimulates osteoclast activation, differentiation, and survival by binding to its receptor [36,37]. OPG is a decoy receptor and an antagonist of RANKL, derived primarily from cells of the osteoblast lineage [38,39]. Our results presented in Figure 6, demonstrated that there were no differences between the investigated materials in relative RANKL gene expression. However, significant differences were observed in regions of the mandibulae, notably in the molar area compared to premolar and canine in all three used materials. Observed differences might be explained by regional specificity of bone tissue in these regions. Relative expression of gene coding OPG was significantly higher in the HAP/PLGA and HAP/PEI groups compared to BioOss. RANKL/OPG ratio can provide an interpretation of the tissue remodeling process and this parameter was significantly higher in the BioOss group compared to HAP/PLGA and HAP/PEI groups. These results in the BioOss group suggest RANKL predominant activity, and, as a consequence, bone resorption. On the contrary, lower values and RANKL/OPG balance in the HAP/PEI group suggest osteocyte homeostasis and maintaining the regulation of bone resorption and bone strength [15,16,17,18].

Furthermore, the observed regional differences (considering the site of application of new materials) may be of potential interest as a checkpoint for planning the interventions that involve the application of evaluated materials in different mandibular regions. Clinical and prognostic relevance of the observed regional differences, including the underlying mechanisms, should be additionally estimated.

## 4. Materials and Methods

### 4.1. Scaffold Preparation and Characterization

The preparation of scaffolds HAP/PLGA and HAP/PEI was performed in ALBOS d.o.o., Belgrade, Serbia using the method of polymer foam template in three steps as reported in previous papers [3,8,40]. Detailed materials characterization is presented in Supplementary file. The first step was the synthesis of HAP powder, used for the synthesis of porous HAP granules in the second step. The third step included the deposition of a thin PLGA and PEI film onto the surface of the granules.

1. Hydrothermal synthesis of HAP powder: The first stage of scaffold synthesis was the hydrothermal synthesis of HAP powder from a stoichiometric mixture of (NH_4_)_2_HPO_4_ and Ca(OH)_2_. After mixing the same volumes of aqueous solutions (500 mL of 3.02 cmol), the hydrothermal treatment was performed in autoclave at 150 °C under the pressure of 5·105 Pa for 8 h. The precipitate was decanted, dried at 80 °C for 48 h, washed with deionized water, and centrifuged. Then, 5 g of hydrothermally synthesized HAP and 1.5 g of poly(ethylene vinyl acetate)/poly(ethylene vinyl versatate) were mixed and further processed in the autoclave at 120 °C for 2 h.
2. Synthesis of porous HAP granules: The obtained HAP powder was mixed with water to form ceramic slurry. Polyurethane foams with the required pore size were then dipped into the slurry to form scaffold porous structure. The ceramic slurry-coated polyurethane foams were left to dry at room temperature, then heated in an oven at 600 °C to burn away the foam, and finally sintered at 1200 °C for 4 h. The obtained porous HAP compact was further disintegrated into granules of sizes 300 µm^−1^ mm.
3. The deposition of a PLGA and PEI film onto the surface of HAP granules: The final step in scaffold synthesis was the deposition of a thin film of PLGA or PEI onto the surface of HAP granules. PLGA pellets were dissolved in chloroform to obtain a 1% w/w solution, which was then poured over the HAP granules. After the solvent evaporation, thin PLGA film was formed on granule surfaces to form HAP/PLGA scaffold (signed in previous reports as ALBO OS). Branched PEI (3 g) was dissolved in 15 mL water by heating and stirring. To reduce amino content and cytotoxicity of PEI it was modified with carbon dioxide (CO_2_). Carbon dioxide was bubbled into this solution at ambient temperature and stirring was continued for 5 h until the reaction was complete. The contents were transferred to an Eppendorf tube, freeze dried to form solid PEI- CO_2_, and later dissolved in ethanol. HA/PEI coatings were obtained by immersion of HAP granules in the prepared solution.

### 4.2. Investigations on Animal Models

In this study domestic swine’s mandibles were used for all implantation procedures. Experiments were approved by the Ethics Committee of the Faculty of Medicine in Kosovska Mitrovica, University of Pristina, Pristina, Kosovo (no. 09-3176) and were performed according to ISO 10993-2:2006 Animal Welfare Requirements [41]. The total number of the animals used was 15, out of which 10 males and 5 females. The pigs were 3 months old at the beginning of the experimental protocol and 7 months old at the end of the protocol. Body weight of the pigs was 20–25 kg at the beginning, and approximately 120 kg at the end of the experiment. Only clinically healthy pigs were taken for the investigations. The health status of the animals was monitored daily during the experiment. The animals were kept in standard cages, one animal per cage, placed in a room where the temperature was held at 22 ± 4 °C and relative humidity at 55 ± 10%. The animals were given ad libitum access to standard food and water.

### 4.3. Surgical Procedure

Surgical procedures were performed in laboratory of the Agricultural School Pristina in Lesak, Serbia. Dissociative anesthesia was conducted in all surgical procedures. The pigs were premedicated by intramuscular administration of Xylazine (2% Xylazine, 5 mg/kg body weight), followed by intramuscularly administered Ketamine (500 mg/mL, 35 mg/kg body weight) and Acepromezine (0.75 mg/kg body weight). The surgical procedure was performed under aseptic conditions in a manner that ensures minimal trauma. Before the intraoral incision, local anesthetic was administered (2 mL articaine with 1:200,000 adrenaline). The intraoral incision was made in the lower vestibule from canine to molar region. Mucoperiosteal flap was elevated and mandibular bone was exposed. Minding the position of the teeth and mandibular canal, three critical size defects (size 10 × 5 mm) were made on each side of the mandible using a trepan borer (AC Dental Implant System, trepan borer 6.0 mm, total length 32 mm, blade length 15.8 mm, inner diameter 6 mm, outer diameter 6.95 mm, titanium alloy), with continuous flushing with saline solution. Three defects were made on each side of the mandible, in the canine, premolar, and molar area. The bone tissue was removed with a sharp excavator. The defects were then filled with either HAP/PLGA, HAP/PEI, or BioOSS**^®^** Geistlich (Wolhusen, Switzerland), using double blind procedure. The same procedure was carried out on both the halves of the mandible. Using this method, a total of 90 mandibular defects were made and three experimental groups were formed:

1. HAP/PLGA group—defects filled with HAP/PLGA scaffold (*n* = 30)
2. HAP/PEI group—defects filled with HAP/PEI scaffold (*n* = 30)
3. Control group—defects filled with BioOss scaffold (*n* = 30)

The mucoperiosteal flap was then returned to its place to cover the experimental area and was stitched up with resorbable sutures. Postoperatively, the animals were injected subcutaneously with analgesics and antibiotics for five days. The animals were placed in individual boxes until the end of the experiment. All animals recovered well after the surgical procedures without any signs of infection. After the assessment period of 4 months, the animals were sacrificed by intravenous injection of pentobarbital and mandibles were collected for further analysis.

### 4.4. Analysis of Bone Density with Cone-Beam Computed Tomography (CBCT)

**Figure 9:**
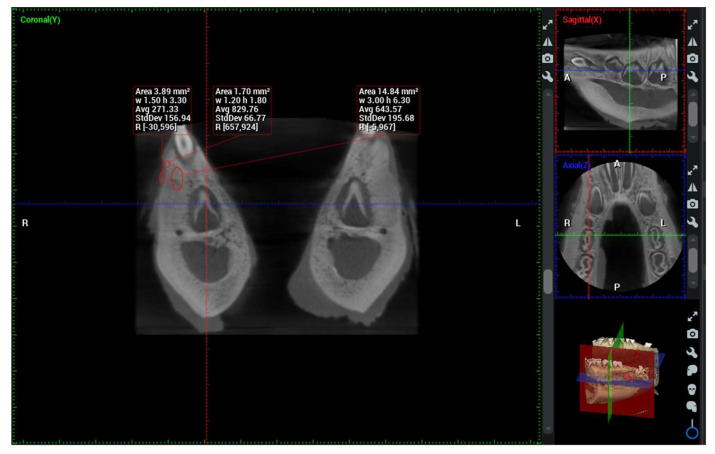
Analysis of bone density with CBCT and regions of interest (ROIs).

Swine mandibles were removed and soft tissue were cleaned. Before radiological assessment, target spots for measurements were marked with fine borer so the same area would be analyzed both radiologically and histologically. Radiological assessment was done by CBCT apparatus (Planmeca Romexis 5.3.4.39, Helsinki, Finland). Imaging parameters selected in all the animals were: slice thickness 0.075 mm; tube voltage 110 kVp; tube current (in mA) and exposure time (in seconds) depending on the volume exposed. Tree-dimensional (3D) images were acquired and analyzed using multiple-planar reconstruction tool of Planmeca Romexis Software (Planmeca Romexis 5.3.4.39, Helsinki, Finland). Axial, sagittal, and transversal multiplanar images were generated to assess the quality of the restored defect and surrounding healthy bone. For quantitative bone density measurements, CBCT grayscale values were measured on the slice images and converted to Hounsfield Units (HU) using the scanner’s software. For the measurement of bone density of operative defect region of interest (ROI) in the sagittal and axial plane, ROI plane was placed in the center of defect, while for the transverse plane ROI comprised defect volume with minimally 1 mm distance from the margins of defect. For the measurement of density of the bone surrounding the defect, ROI comprised periphery of the defect to the normal bone with 3 mm thickness. Similar measurements were performed to measure bone density of normal mandibular bone ensuring that roots of the teeth were not in the ROI. The values from the three planes were averaged in order to reduce error associated with measurement technique. Results were reported as bone density in the operative defect, bone immediately surrounding the defect and normal surrounding bone expressed in HU (Figure 9).

### 4.5. Histological Analysis

To prepare the decalcified histological sections, formalin fixed-tissue specimens were soaked in 10% formic acid solution. The solution was changed once in 3 days and the pH and temperature were recorded on a daily basis. After ensuring complete decalcification, the tissues were washed using distilled water for 30 min, following which the specimens were subjected to automatic tissue processing. Specimens were dehydrated in a graded ethanol series and xylene and were embedded in paraffin. The paraffin blocks were cut into 5–7 µm thickness using a microtome and then attached to adhesive slides. The tissue specimens were stained with hematoxylin-eosin and Picrosirius red staining solutions and photographed by optical microscope (Olympus BX, Tokyo, Japan) equipped with a digital camera. For quantitative analysis of collagen, bright field images of Picrosirius red stained sections were captured at 20× magnification, and the positive areas in visual fields, were measured using ImageJ software (National Institute of Health, Bethesda, MD, USA). Total number of cells in the region of interest (bone surface) was calculated using ImageJ software. Scoring and histological analysis were performed in blinded fashion by two independent observers. For each investigated region, we analyzed five fields per section. The results are presented as mean count of cells per bone surface or mean count of visual field (area) percentage.

For immunohistochemical analysis, slices with 5 μm-thicknesses were taken from the middle of each bone specimen sections. The slices were deparaffinized, rehydrated, and heat-treated for antigen retrieval. Hydrogen peroxide (3%) was used to block the activity of endogenous peroxidase. Immunohistochemical staining was performed by incubating deparaffinized bone tissue sections with primary mouse anti—osteocalcin antibody (5 mg/mL, NBP2-89037, Novus Biologicals, Englewood, CO, USA) overnight at room temperature. Staining was visualized by using Expose mouse and rabbit specific HRP/DAB detection IHC Kit (ab80436, Abcam, Cambridge, UK). The sections were counterstained with Mayer’s hematoxylin and photomicrographed by a light microscope (Olympus BX51, Tokyo, Japan) equipped with a digital camera. Results are presented as a mean count of immunoreactive staining score. Immunohistochemical findings were semi-quantitatively evaluated as follows: 0 =  negative; 1 =  weak; 2 =  moderate; 3 =  strong; 4 = very strong [42].

### 4.6. RT PCR Analysis

Bone specimens were excised and snap frozen in liquid nitrogen prior to homogenization. Total RNA from the bone specimens was extracted using TRIzol reagent (Invitrogen, Waltham, MA, USA) according to the manufacturer’s instructions. For reverse transcription, iScript Re-verse Transcription Mastermix (Bio-Rad, Hercules, CA, USA) was used. Real-time PCR was carried out using SsoAdvanced Universal SYBR Green Supermix (Bio-Rad, Hercules, CA, USA) according to the manufacturer protocol, and mRNA-specific primers (Table 1) for OPG, RANKL and β-actin as a housekeeping gene (Invitrogen, Waltham, MA, USA). All samples underwent the same RT-PCR protocol: activation at 95 °C for 30 s followed by 40 cycles including denaturation at 95 °C for 10 s, annealing and extension at 60 °C for 30 s. Quantitative RT-PCR reactions were done in the Applied Biosystems 7500 (Applied Biosystems, Waltham, MA, USA). Quantitative RT-PCR reactions were performed in duplicate, and the mean values were further analyzed. After data analysis, relative gene expression was calculated according to Livak and Schmittgen [43].

### 4.7. Statistical Analysis

Statistical analysis was performed by using the SPSS version 20.0 statistical package (IBM SPSS Statistics 20, Chicago, MA, USA). The results are expressed as the means ± standard errors of the mean (SEM). Parameters were initially submitted to the Levene’s test for homogeneity of variance and to the Shapiro–Wilk test of normality. One-way ANOVA, followed by Bonferroni test was used for comparisons between the groups. Kruskal–Wallis, followed by Dunn’s test was used as nonparametric test were appropriate. Contingency tables were used for analysis of data obtained by immunohistochemistry. The significance was determined at *p* < 0.05 for all analyses.

## 5. Conclusions

The results obtained in this study provided the evidence that composite scaffolds (HAP/PLGA and HAP/PEI) demonstrated improved biological behavior after implantation compared to conventional allograft in treatment of swine’s mandibular defects. HAP/PEI composite scaffold was notably better in terms of bone density and bone tissue histological characteristics. Furthermore, the observed differences considering the site of application of new materials may be of potential interest as a checkpoint for planning the interventions that involve the application of evaluated materials in different mandibular regions. Clinical and prognostic relevance of the observed regional differences should be further investigated.

## Figures and Tables

**Table 1:** RT-PCR primers used in this study.

Target Gene	Forward	Reverse	Genbank Accession No.
β-actin	TTGCTGACAGGATGCAGAAG	GAGCCTCCAATCCAGACAGA	ABF19863.1
OPG	CCAAGGTATCGACCTCTGTGA	GGGCAAGCTTTGCATTAAGA	XM_003481346.4
Osteocalcin	GAAGAGACTCAGGCGCTACC	GGGTTGAGCTCACACACCTC	NM_001164004.1
RANKL	ACACGGATTTGCAAGACACA	CTGCATTTCCTTTTGCACAG	XM_001925694.6

## Data Availability

The data presented in this study are available on request from the corresponding author.

## Supplementary Materials

The following are available online at, Supplementary file: Brief structural and physico-chemical properties of scafold materials.
