# Supplementary material for: Comparison of Hydroxyapatite/Poly(lactide-co-glycolide) and Hydroxyapatite/Polyethyleneimine Composite Scaffolds in Bone Regeneration of Swine Mandibular Critical Size Defects: In Vivo Study
Source: Molecules. 2022 Mar 4;27(5):1694. doi: 10.3390/molecules27051694 (PMC8911599; doi:10.3390/molecules27051694)
Supplement: Supplementary file 1 [file molecules-27-01694-s001.zip › molecules-1602488-supplementary.pdf]

Supplementary Information

Physico-chemical and structural characterization of the hydroxyapatite (HAP) powder, the starting component for the synthesis of HAP granules - the main components of HAP/PLGA and HAP/PEI scaffold and xenograft BioOssGeistlich

1. CHARACTERIZATION METHODS

1.1. For the structural analysis of the HAP powder, which is used as starting component for production of HAP porous granules, X-ray diffraction (XRD) and Infrared Spectroscopy were applied. For XRD analysis diffractometer Philips PW 1050 was used with its Cu-Kα1-2 lamp, wherein the data were acquired in the range of 2θ from 9 to 67°, in steps of 5°, and an exposure time of 2 seconds per step. For infrared analysis the FT-IR spectrometer FTIR Nicollet 380, Thermo Electron Corporation was used. IR spectra were taken in the spectral range from 4000 to 400 cm^{-1}.

The obtained XRD spectrum shows that the hydrothermally synthesized HAP powder corresponds to a carbonated calcium hydroxyapatite $Ca_{10}(PO_4)_6(OH)_2$ (JCPDS 9-432). All characteristic diffraction peaks are present: (211) at 2θ = 31.9°; (112) at 2θ = 32.26°, (300) at 2θ = 33.12°, (002) at 2θ = 25.86°, (222) at 2θ = 46.86° and (213) at 2θ = 49.58°. The crystallite size, calculate dusing Scherer formula was 8-22 nm.

IR spectrum of the HAP powder shows bands typical for HAP. Bands at about 1092, 1042, 957, 603, 569 and 473 cm^{-1} correspond to PO_4^{3-} group, which is a part of HAP. The vibrations of the OH groups present in the HAP, were detected at about 630 and 1626 cm^{-1}. CO_3^{2-} group vibrations at 1442, 1406 and 875 cm^{-1} indicate the presence of the carbonate groups in the apatite structure, which partially replaced OH^- ions. The obtained apatite is identified as a carbonate HAP type B, which is the most active form of carbonate apatite (prevails in the bone of young people).

1.2. Microstructure and surface properties of scaffolds

Scanning electron microscopy (SEM) and atomic force microscopy (AFM) were used to analyze the microstructure and surface properties of HAP and Bio-Oss.

SEM examinations of HAP/PLGA and HAP/PEI scaffolds show a well-defined internal geometry, with pores of diameters from 100 to 130 nm, and grains of mean diameters from 100 to 220 nm. Regarding Bio-Oss SEM show irregular internal geometry with grains of mean diameters from 50 to 150 nm and pores of diameters from 20 to 100 nm (Figure S1)

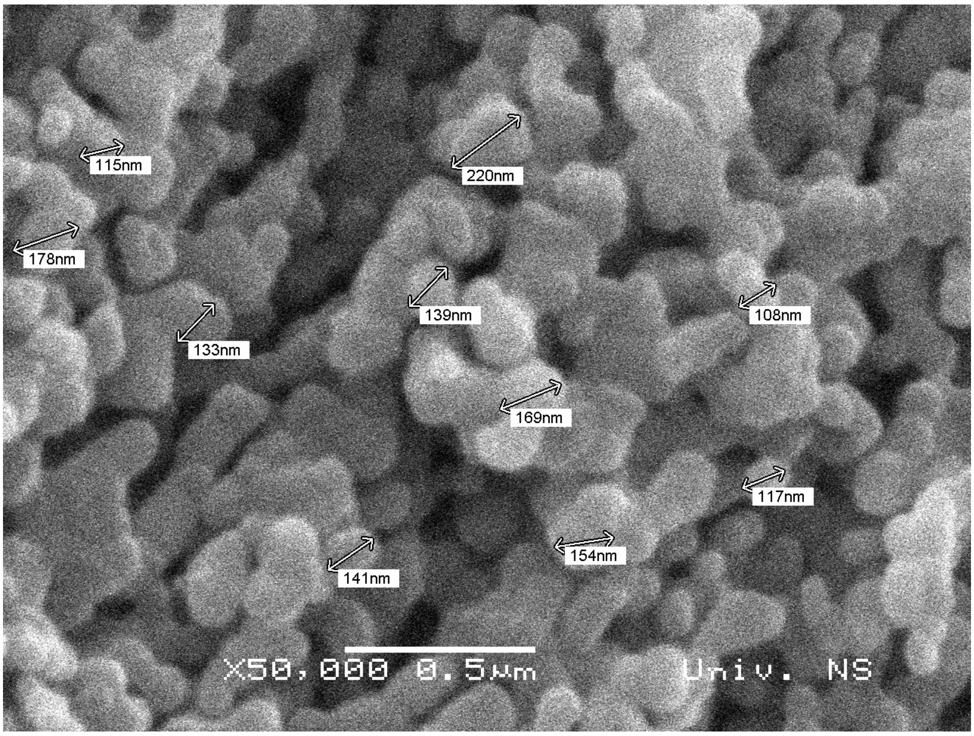

Figure S1. SEM micrograph of HAP/PLGA scaffold

AFM images of HAP/PLGA and HAP/PEI appear typically as a comb-like wall structure with channels of pores between the walls. The channels dimensions are: length ~10–30 µm, width ~1 µm (Figure S2). In the case of BioOss, nano-patterns on the walls are also visible. Channels of pores, with length >680 nm and width 60–190 nm, are irregularly shaped.

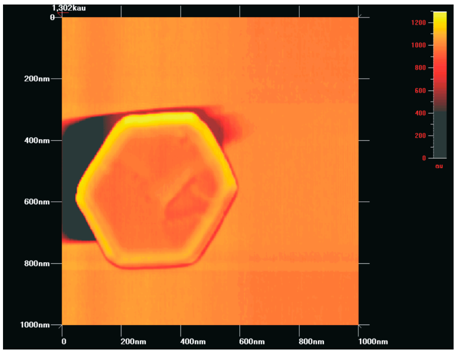

Figure S2. AFM image of HAP/PLGA scaffold.

1.3. Scaffold porosity

HAP/PLGA, HAP/PEI and Bio-Oss samples were scanned using micro-computed tomography (CT) (Skyscan-Bruker 1172, Kontich, Belgium) with a source voltage of 100 kV, a current of 100 μA, exposure time of 1000 ms and copper aluminum filter. The slice thickness was 5 μm isotropic resolution and 2048×2048 pixels per slice. Cross-sectional slices were reconstructed using the NRecon v.1.6.9.8 software with beam hardening correction of 10%, ring artifact correction of 9%, postaligment of -2.5 and smoothing of 2. CTAn 1.14.4.1 software (SkyScan-Bruker) with threshold set to 66–256 values was employed to distinguish scaffold and air. Porosities of investigated scafdfolds were additionally determined by high-pressure mercury intrusion porosimetry (Carlo Erba Porosimeter 2000 using Milestone 100 Software System).

Micro CT image shows a very high porosity of HAP as the main constituent of HAP/ PLGA and HAP/PEI scaffolds. There is very high percentage of open porosity, volume partition of closed pored is negligible (Figure S3). The connectivity density (number of connected pores per mm3) is high. Porosity of BioOss showed significant differences indicating that granules have different densities, which is expected for the natural bone. Average values for open and total porosity were lower than in HAP/ PLGA and HAP/PEI scaffolds.

The most important parameter of the scaffold structure is the pore size distribution. Pore size distribution in HAP/ PLGA and HAP/PEI scaffolds is much wider than in BioOss. Maximal pore size in Bio-Oss is 330 μm, while in HAP/ PLGA and HAP/PEI scaffolds it is 774 μm. In

Bio-Oss, pores with the lowest diameters are more frequent than in HAP/ PLGA and HAP/PEI scaffolds.

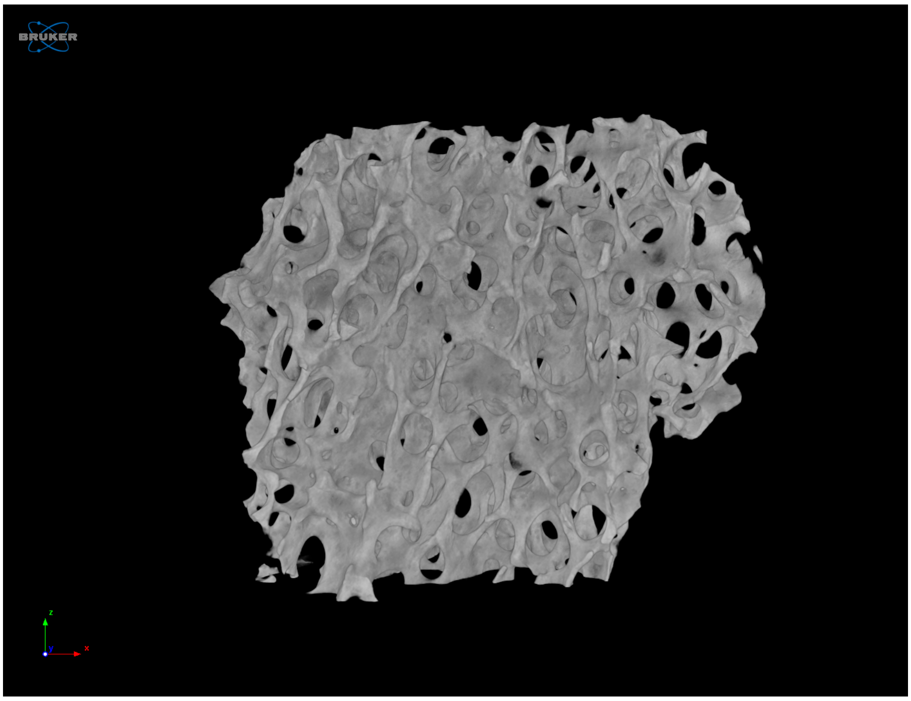

Figure S3. Micro-CT image of HAP scaffold.

Nano-porosity of HAP/ PLGA and HAP/PEI scaffolds and BioOss obtained by mercury porosimetry showed the diameters of nanopores in HAP/ PLGA and HAP/PEI scaffolds in the range 30–400 nm, with the highest partition of the pores of 115 nm in diameter, while in Bio-Oss nano-pores are in the range 5–1000 nm wherein the pores of 25 nm are the most dominant.

1.4. Material degradation

The degradation rates of HAP granules and Bio-Oss were determined after their immersion in phosphate-buffered saline (PBS) with added Tris buffer at pH 7.4. The degradation rate based on the change of Ca^{2+} concentrations in the medium was measured by atomic absorption spectroscopy (Perkin Elmer 3030B). For each measurement, an aliquot of 3 ml of the medium was taken and replaced by the same volume of fresh PBS with Tris buffer. The measurements were performed 10 and 30 min, 1, 4 and 8 hours, 1, 2, 5, 8, 12, 15, 19 and 22

days after the immersion. The obtained value for HAP/PLGA and HAP/PEI were much lower than that of BioOss indicating higher degradation rate of HAP.
